# *MAD1L1* and *TSNARE* gene polymorphisms are associated with schizophrenia susceptibility in the Han Chinese population

**DOI:** 10.1186/s12920-021-01070-2

**Published:** 2021-09-04

**Authors:** Xianglai Liu, Hailing Xie, Zejuan Fu, Qiankun Yao, Tianming Han, Dafei Zhan, Zhan Lin, Hong Zhu

**Affiliations:** 1 Institute of Mental Health, Hainan Provincial Anning Hospital, No 10, Nanhai Avenue East, Haikou, 571100 Hainan China; 2 First Department of Psychiatry, Hainan Provincial Anning Hospital, Haikou, 571100 Hainan China; 3 Department of Nursing, Hainan Provincial Anning Hospital, Haikou, 571100 Hainan China; 4 Department of Prevention Section, Hainan Provincial Anning Hospital, Haikou, 571100 Hainan China

**Keywords:** Schizophrenia, *MAD1L1*, *TSNARE*, Susceptibility

## Abstract

**Background:**

Schizophrenia (SCZ) is a severe mental illness with high heritability. This study aimed to explore the correlation between *MAD1L1*, *TSNARE* polymorphisms and SCZ susceptibility.

**Methods:**

A total of 493 SCZ patients and 493 healthy controls were included. The genotypes of *MAD1L1* and *TSNARE* polymorphisms were identified by Agena MassARRAY platform. Odds ratio (OR) and 95% confidence intervals (CIs) were tested via logistic regression analysis in multiple genetic models and different subgroups.

**Results:**

We observed that AG genotype of rs1107592, AG genotype of rs4976976, and CA genotype of rs67756423 decreased the susceptibility to SCZ (*p* < 0.05). Age stratification analysis showed that the TC genotype of rs12666575, AG genotype of rs1107592, and AG genotype of rs4976976 decreased the risk of SCZ individuals older than 36 years (*p* < 0.05). In addition, the AG and AA genotype of rs4976976, the CA genotype of rs67756423 were associated with a lower risk of SCZ in males (*p* < 0.05). In females, the TT genotype of rs12666575 in recessive model, the AG and AA-AG genotype of rs1107592 in heterozygote and dominant model, could reduce the susceptibility to SCZ (*p* < 0.05). However, no significant association was found after Bonferroni correction.

**Conclusions:**

Our results suggest that *MAD1L1* and *TSNARE* genetic polymorphisms exert a protective role in the risk of SCZ. These findings provide evidence that *MAD1L1* and *TSNARE* may serve as potential biomarkers of SCZ. However, a replication experiment in a cohort with large sample size are required to confirm our findings.

*Trial registration* Not applicable.

**Supplementary Information:**

The online version contains supplementary material available at 10.1186/s12920-021-01070-2.

## Background

Schizophrenia (SCZ) is a mental illness characterized by hallucinations, delusions, emotional disorders, and social withdrawal [1]. SCZ causes some suffering for individuals and poses a huge psychosocial and economic burden to families and societies. Saha et al. reported a median incidence of 15.2 per 100,000 persons and a lifetime prevalence of 0.4–1% of the general population [2]. In 2010 there were 7.16 million people in China affected by SCZ during their lifetime, an increase of 132% compared with 1990. And the prevalence of SCZ in urban China was 0.39% in 1990, 0.57% in 2000, and 0.83% in 2010 [3]. However, the pathogenesis of SCZ is not clear. Recently, many studies have indicated that genetic factors were important in the development of SCZ [4, 5]. And Cardno et al. reported that the heritability of SCZ is up to 80% [6]. Previous genetic studies have identified some candidate genes (AKT1, 5-HTT, COMT) as risk genes for SCZ to illustrate the biological mechanism of this disorder [5]. However, the exact roles of these candidate genes in SCZ pathogenesis were not fully established.

Mitotic arrest deficient-like 1 (*MAD1L1*) is a component of the mitotic spindle-assembly checkpoint which prevents the onset of anaphase until all the chromosomes are properly aligned at the metaphase plate [7]. *MAD1L1* involved in tumor suppression and cell cycle control. A large body of literature has demonstrated that the expression of *MAD1L1* is abnormal in breast cancer, small-cell lung cancer, and other cancers [8, 9]. Besides, it was found that *MAD1L1* was related to the reward systems functioning in healthy adults [10]. In a recent study found that *MAD1L1* antigene showed increased IgG level in SCZ patients compared with control subjects [11]. Zhao et al. indicated that single nucleotide polymorphism (SNP) in *MAD1L1* was significantly associated with bipolar disorder in Chinese people [12]. Nevertheless, there are few studies on the role of *MAD1L1* variants in SCZ development.

T-SNARE domain-containing 1 gene (*TSNARE1*) may have evolved from the harbinger transposon superfamily within the vertebrate lineage [13]. It has been suggested that *TSNARE* possesses functions related to transcriptional regulation, nuclear import, and DNA binding [14]. Then, bioinformatic predictions indicated it may bind SNARE and have SNAP receptor activity. Additionally, a genome-wide association study (GWAS) meta-analysis has reported that *TSNARE1* rs10098073 and rs4129585 were closely related to SCZ and bipolar susceptibility in Caucasians [15]. This was in line with the discovery of Gu et al., which indicated a significant correlation between rs10098073, rs4129585 in *TSNARE* and SCZ risk in Southeast Chinese Han and Zhuang people [16]. However, the relationship between other SNPs polymorphisms in *TSNARE* and SCZ susceptibility has not been explored in the Northwest Chinese Han population.

In the present study, we mainly focused on the role of *MAD1L1* and *TSNARE1* in the pathogenesis of SCZ. We evaluated the association of *MAD1L1* rs10275045, rs12666575, rs1107592 and *TSNARE1* rs4976976, rs67756423 with SCZ risk in the Northwest Chinese Han population. These findings will provide insights into the pathogenesis and development of SCZ.

## Methods

### Study subjects

A total of 986 individuals, which included 493 SCZ patients and 493 controls, were enrolled from Xi’an Mental Health Center. Schizophrenia was identified by two psychiatrists on the basis of the Tenth Revision of International Classification of Diseases. Patients who met the following conditions were excluded: (1) mental diseases induced by organic brain syndrome, (2) neurological diseases, (3) mental retardation, (4) severe brain injury, (5) non-cooperating patients with superexcitation, (6) pregnant or breastfeeding women. Inclusion criteria for healthy controls were individuals without family history of mental disorder, severe head injury, febrile convulsion in childhood or infant stage. Moreover, we used G*power software to calculate the minimal required sample size based on the probability of a typeIerror of alpha = 5%, typeIIerror of beta = 15% (power = 85%), effect size of 0.2. This calculation yielded a sample consisting of at least 450 cases and 450 controls. Then, we recruited 493 cases and 493 controls in this study.

The legal guardian of these participants provided informed consent documents on their behalf. This study got approval of the Ethics Committee of Xi’an Mental Health Center and followed the Declaration of Helsinki.

### SNP genotyping

Peripheral blood samples were collected from each subject. DNA was isolated from venous blood sample by the GoldMag DNA purification kit (GoldMag Co. Ltd, Xi′an, China) in accordance with the user’s protocol, then quantified by NanoDrop 2000 (Thermo Scientific, Waltham, MA, USA). The SNPs in the *MAD1L1*, *TSNARE1* genes were chosen based on the minor allele frequency (MAF) > 0.05 in Han Chinese from the 1000 Genome Projects. Three SNPs (rs10275045, rs12666575, rs1107592) in *MAD1L1* and two SNPs (rs4976976, rs67756423) in *TSNARE1* were selected in the present study.

Primers of the five SNPs are listed in Additional file 1: Table S1. PCR reactions were performed in a buffer containing 1 μl DNA, 0.5 μl PCR Buffer, 0.4 μl MgCl_2_, 0.1 μl dNTP Mix, 1.0 μl primer mix, and 0.2 μl Taq ligase in a final reaction volume of 5 μl. The reaction mixture was heated to 94 °C for 15 min for denaturation. Then, the sample was subjected to 45 cycles of 94 °C 20 s, annealing at 56 °C 30 s and extension at 72 °C 60 s, followed by a final extension step at 72 °C for 3 min. The PCR product was used to genotype using the Agena MassArray platform (Agena Bioscience, San Diego, CA, USA). The raw data was analyzed and managed using Agena Typer 4.0 software (Agena Bioscience, San Diego, CA, USA).

### Data analysis

We performed Pearson’s χ^2^ test and student’s t-test to assess the differences in gender and age of study populations, respectively. Hardy–Weinberg equilibrium (HWE) was examined by Pearson’s χ^2^ test. The distribution of SNP allele and genotype between SCZ patients and healthy controls were tested by χ^2^ test. Odds ratio (OR) and 95% confidence intervals (CI) were applied to estimate the relationship between *MAD1L1*, *TSNARE1* gene and SCZ risk by logistic regression analysis in multiple inheritance models and subgroup. We also evaluated the SNP-SNP interaction in the risk of SCZ using multifactor dimensionality reduction (MDR). Statistical power and false positive report probability (FPRP) values were calculated by the Excel spreadsheet which was offered on Wacholder’s website [17]. The functional role of these SNPs was predicted by HaploReg database (https://pubs.broadinstitute.org/mammals/haploreg/haploreg.php). The differences were deemed significant at *p* < 0.05, whereas a value of corrected *p* < 0.05/5 was considered significant after Bonferroni correction.

## Results

### Study subjects

Totally, 493 patients (261 men and 236 women) of SCZ and 493 healthy controls (257 men and 232 women) were enrolled with a mean age of 36.47 ± 13.20 years and 36.50 ± 11.89 years, respectively (Table 1). There were no statistical differences in age (*p* = 0.968) and gender (*p* = 0.799) between the two groups.

**Table 1:** Characteristics of cases and controls

Variables	Cases (n = 493)	Controls (n = 493)	*p*
Age, year (mean ± SD)	36.47 ± 13.20	36.50 ± 11.89	0.968^a^
> 36	220 (44.6%)	230 (46.7%)	
≤ 36	273 (55.4%)	263 (53.3%)	
Gender			0.799^b^
Male	261 (52.9%)	257 (52.1%)	
Female	236 (47.1%)	232 (47.9%)	

SD: standard deviation

*p*^a^ values were calculated from student’s t test

*p*^b^ values were calculated from χ^2^ test

*p* < 0.05 indicates statistical difference

### Basic information for the candidate SNPs

Three SNPs (rs10275045, rs12666575, rs1107592) in *MAD1L1* and two SNPs (rs4976976, rs67756423) in *TSNARE1* were successfully genotyped. In Table 2, we described the details of the selected SNPs regarding SNP ID, gene, chromosomal position, role, MAF. All SNPs were following HWE (*p* > 0.05) and were found in the intron region.

**Table 2:** Basic characteristics and allele frequencies of the candidate SNPs in *MAD1L*1 and *TSNARE1*

SNP	Chr	Position	Gene	Role	Allele minor/major	MAF		HWE		HaploReg
						Case	Control	Case	Control	
rs10275045	7	1881190	*MAD1L1*	Intron	T/C	0.435	0.447	0.988	0.315	Motifs changed; NHGRI/EBI GWAS hits; GRASP QTL Hits; Selected eQTL hits
rs12666575	7	1964786	*MAD1L1*	Intron	T/C	0.458	0.456	0.116	0.170	Enhancer histone marks; DNAse; NHGRI/EBI GWAS hits; Selected eQTL hits
rs1107592	7	2001797	*MAD1L1*	Intron	A/G	0.453	0.465	0.029	0.278	Enhancer histone marks; DNAse; Motifs changed; NHGRI/EBI GWAS hits; GRASP QTL Hits; Selected eQTL hits
rs4976976	8	142230292	*TSNARE1*	Intron	A/G	0.505	0.499	0.016	0.207	DNAse; Motifs changed
rs67756423	8	142252164	*TSNARE1*	Intron	C/A	0.360	0.372	0.019	0.147	Selected eQTL hits

SNP: Single nucleotide polymorphism; Chr: chromosome; MAF: Minor allele frequency; HWE: Hardy–Weinberg equilibrium; OR: Odds ratio; 95% CI: 95% confidence interval

*p* values were calculated from χ^2^ test

### SCZ susceptibility evaluation

Multiple inheritance models (allele, codominant, dominant, recessive, and additive models) were performed to assess the relationship between SNPs and SCZ susceptibility (Table 3). We found that individuals carrying the heterozygous genotype AG in rs1107592 (OR = 0.72, 95% CI = 0.54–0.97, *p* = 0.031), AG in rs4976976 (OR = 0.73, 95% CI = 0.54–0.99, *p* = 0.043), CA in rs67756423 (OR = 0.72, 95% CI = 0.55–0.94, *p* = 0.017) were reduced the susceptibility to SCZ when compared with the GG, GG, and AA genotype. However, no significant association was found after Bonferroni correction. Moreover, the significant association of rs10275045 and rs12666575 in *MAD1L1* with SCZ susceptibility was not detected.

**Table 3:** Relationship of polymorphisms in *MAD1L1* and *TSNARE1* genes and SCZ susceptibility

Gene	SNP	Model	Genotype	OR (95% CI)	*p*
*MAD1L1*	rs10275045	Allele	C	1.00	
			T	0.95 (0.80–1.14)	0.589
		Codominant	CC	1.00	
			TT	0.93 (0.64–1.34)	0.680
			TC	0.87 (0.65–1.16)	0.334
		Dominant	CC	1.00	
			TT-TC	0.88 (0.67–1.16)	0.372
		Recessive	TC-CC	1.00	
			TT	1.01 (0.73–1.39)	0.946
		Additive	–	0.95 (0.79–1.14)	0.580
*MAD1L1*	rs12666575	Allele	C	1.00	
			T	1.01 (0.85–1.21)	0.909
		Codominant	CC	1.00	
			TT	1.07 (0.75–1.53)	0.721
			TC	0.78 (0.58–1.05)	0.101
		Dominant	CC	1.00	
			TT-TC	0.86 (0.65–1.13)	0.279
		Recessive	TC-CC	1.00	
			TT	1.24 (0.91–1.70)	0.166
		Additive	–	1.01 (0.84–1.21)	0.920
*MAD1L1*	rs1107592	Allele	G	1.00	
			A	0.96 (0.80–1.14)	0.616
		Codominant	GG	1.00	
			AA	0.96 (0.67–1.37)	0.820
			AG	**0.72** (**0.54–0.97)**	**0.031**
		Dominant	GG	1.00	
			AA-AG	0.79 (0.60–1.04)	0.093
		Recessive	AG-GG	1.00	
			AA	1.17 (0.86–1.59)	0.308
		Additive	–	0.96 (0.80–1.14)	0.625
*TSNARE1*	rs4976976	Allele	G	1.00	
			A	1.03 (0.86–1.22)	0.787
		Codominant	GG	1.00	
			AA	1.05 (0.74–1.49)	0.801
			AG	**0.73 (0.54–0.99)**	**0.043**
		Dominant	GG	1.00	
			AA-AG	0.83 (0.62–1.10)	0.192
		Recessive	AG-GG	1.00	
			AA	1.29 (0.97–1.72)	0.083
		Additive	–	1.02 (0.86–1.22)	0.790
*TSNARE1*	rs67756423	Allele	A	1.00	
			G	0.95 (0.79–1.14)	0.576
		Codominant	AA	1.00	
			CC	1.10 (0.74–1.62)	0.641
			CA	**0.72 (0.55–0.94)**	**0.017**
		Dominant	AA	1.00	
			CC-CA	0.79 (0.62–1.03)	0.077
		Recessive	CA-AA	1.00	
			CC	1.31 (0.91–1.88)	0.151
		Additive	–	0.95 (0.79–1.14)	0.587

SNP: single nucleotide polymorphism; OR, odds ratio; 95% CI, 95% confidence interval

*p* values were calculated by logistic regression analysis with adjustments for age and gender

Bold values indicate statistical significance (*p* < 0.05)

Subsequently, we carried out stratification analysis based on age and gender (Table 4). Among people older than 36 years, the TC heterozygote in rs12666575 (OR = 0.64, 95% CI = 0.41–1.00, *p* = 0.048), the AG heterozygote in rs1107592 (OR = 0.62, 95% CI = 0.40–0.96, *p* = 0.034), and the AG heterozygote in rs4976976 (OR = 0.63, 95% CI = 0.40–0.99, *p* = 0.045) had a risk-decreasing effects compared with the CC, GG and GG homozygote. However, no significant association was found after Bonferroni correction.

**Table 4:** Relationships of *MAD1L1* and *TSNARE1* polymorphisms with SCZ risk stratified by age and gender

Gene SIP	Model	Genotype	≤ 36		> 36		Male		Female	
			OR (95% CI)	*p*	OR (95% CI)	*p*	OR (95% CI)	*p*	OR (95% CI)	*p*
*MAD1L1* rs10275045	Allele	C	1.00		1.00		1.00		1.00	
		T	0.91 (0.71–1.16)	0.429	1.01 (0.78–1.32)	0.937	0.85 (0.67–1.09)	0.201	1.08 (0.83–1.40)	0.575
	Codominant	CC	1.00		1.00		1.00		1.00	
		TT	0.81 (0.50–1.33)	0.404	1.05 (0.60–1.83)	0.878	0.74 (0.44–1.24)	0.250	1.16 (0.69–1.97)	0.572
		TC	1.03 (0.70–1.52)	0.883	0.66 (0.43–1.03)	0.065	0.70 (0.47–1.05)	0.082	1.08 (0.71–1.63)	0.730
	Dominant	CC	1.00		1.00		1.00		1.00	
		TT-TC	0.96 (0.67–1.39)	0.838	0.75 (0.49–1.13)	0.171	0.71 (0.48–1.04)	0.079	1.10 (0.74–1.63)	0.634
	Recessive	TC-CC	1.00		1.00		1.00		1.00	
		TT	0.80 (0.52–1.23)	0.304	1.36 (0.84–2.21)	0.207	0.93 (0.60–1.45)	0.747	1.11 (0.70–1.77)	0.649
	Additive	–	0.92 (0.72–1.17)	0.477	0.97 (0.74–1.28)	0.855	0.84 (0.65–1.08)	0.179	1.08 (0.83–1.40)	0.568
*MAD1L1* rs12666575	Allele	C	1.00		1.00		1.00		1.00	
		T	0.98 (0.77–1.24)	0.853	1.05 (0.81–1.37)	0.707	0.95 (0.74–1.22)	0.691	1.08 (0.83–1.40)	0.566
	Codominant	CC	1.00		1.00		1.00		1.00	
		TT	0.99 (0.61–1.61)	0.974	1.17 (0.68–2.02)	0.563	0.92 (0.56–1.50)	0.728	1.29 (0.76–2.18)	0.342
		TC	0.91 (0.61–1.35)	0.645	**0.64 (0.41–1.00)**	**0.048**	0.86 (0.57–1.30)	0.472	0.70 (0.46–1.06)	0.093
	Dominant	CC	1.00		1.00		1.00		1.00	
		TT-TC	0.93 (0.64–1.36)	0.722	0.77 (0.51–1.16)	0.210	0.88 (0.60–1.29)	0.504	0.83 (0.56–1.24)	0.371
	Recessive	TC-CC	1.00		1.00		1.00		1.00	
		TT	1.05 (0.69–1.60)	0.817	1.55 (0.97–2.48)	0.064	1.01 (0.66–1.54)	0.975	**0.61 (0.52–1.15)**	**0.042**
	Additive	–	0.99 (0.78–1.26)	0.923	1.04 (0.79–1.36)	0.791	0.95 (0.74–1.22)	0.682	1.08 (0.83–1.40)	0.560
*MAD1L1* rs1107592	Allele	G	1.00	0.669	1.00		1.00		1.00	
		A	0.95 (0.75–1.21)		0.97 (0.75–1.26)	0.809	1.08 (0.84–1.37)	0.561	0.84 (0.65–1.09)	0.185
	Codominant	GG	1.00		1.00		1.00		1.00	
		AA	0.92 (0.57–1.49)	0.731	0.99 (0.59–1.69)	0.980	1.20 (0.74–1.94)	0.452	0.73 (0.43–1.23)	0.239
		AG	0.82 (0.55–1.21)	0.312	**0.62 (0.40–0.96)**	**0.034**	0.82 (0.55–1.22)	0.325	**0.62 (0.40–0.95)**	**0.029**
	Dominant	GG	1.00		1.00		1.00		1.00	
		AA-AG	0.85 (0.59–1.22)	0.376	0.72 (0.47–1.09)	0.120	0.93 (0.64–1.34)	0.691	**0.65 (0.43–0.98)**	**0.038**
	Recessive	AG-GG	1.00		1.00		1.00		1.00	
		AA	1.04 (0.68–1.58)	0.860	1.35 (0.86–2.11)	0.193	1.35 (0.89–2.06)	0.157	1.00 (0.6–1.56)	0.992
	Additive	–	0.94 (0.74–1.20)	0.635	0.97 (0.75–1.26)	0.827	1.07 (0.84–1.36)	0.580	0.84 (0.64–1.09)	0.179
*TSNARE1* rs4976976	Allele	G	1.00		1.00		1.00		1.00	
		A	0.98 (0.77–1.24)	0.859	0.92 (0.71–1.20)	0.540	1.04 (0.81–1.33)	0.754	1.10 (0.85–1.42)	0.472
	Codominant	GG	1.00		1.00		1.00		1.00	
		AA	0.97 (0.60–1.55)	0.884	0.85 (0.50–1.44)	0.551	1.10 (0.67–1.80)	0.715	1.19 (0.72–1.97)	0.490
		AG	0.73 (0.48–1.10)	0.136	**0.63 (0.40–0.99)**	**0.045**	**0.61 (0.40–0.93)**	**0.022**	0.96 (0.62–1.50)	0.865
	Dominant	GG	1.00		1.00		1.00		1.00	
		AA-AG	0.80 (0.54–1.18)	0.268	0.70 (0.46–1.07)	0.095	0.74 (0.49–1.10)	0.136	1.04 (0.69–1.57)	0.852
	Recessive	AG-GG	1.00		1.00		1.00		1.00	
		AA	1.19 (0.80–1.75)	0.397	1.15 (0.74–1.78)	0.537	**0.53 (0.42–0.89)**	**0.041**	1.22 (0.81–1.84)	0.340
	Additive	–	0.98 (0.77–1.24)	0.868	0.92 (0.70–1.19)	0.507	1.04 (0.82–1.33)	0.742	1.09 (0.85–1.40)	0.488
*TSNARE1* rs67756423	Allele	A	1.00		1.00		1.00		1.00	
		C	0.97 (0.75–1.24)	0.787	0.93 (0.71–1.22)	0.600	0.90 (0.69–1.16)	0.399	1.01 (0.78–1.32)	0.931
	Codominant	AA	1.00		1.00		1.00		1.00	
		CC	1.13 (0.67–1.91)	0.649	1.04 (0.57–1.89)	0.896	1.05 (0.60–1.85)	0.854	1.15 (0.67–2.00)	0.609
		CA	0.71 (0.49–1.02)	0.065	0.71 (0.48–1.07)	0.106	**0.64 (0.44–0.92)**	**0.017**	0.82 (0.55–1.22)	0.321
	Dominant	AA	1.00		1.00		1.00		1.00	
		CC-CA	0.79 (0.56–1.12)	0.190	0.78 (0.53–1.14)	0.199	0.71 (0.50–1.01)	0.056	0.89 (0.61–1.30)	0.548
	Recessive	CA-AA	1.00		1.00		1.00		1.00	
		CC	1.35 (0.83–2.20)	0.229	1.25 (0.71–2.17)	0.441	1.33 (0.79–2.26)	0.282	1.29 (0.77–2.14)	0.333
	Additive	–	0.96 (0.75–1.23)	0.749	0.93 (0.70–1.22)	0.587	0.89 (0.69–1.15)	0.390	1.01 (0.78–1.31)	0.937

SNP, single nucleotide polymorphism; OR, odds ratio; 95% CI, 95% confidence interval

*p* values were calculated by logistic regression analysis with adjustment for age and gender

Bold values indicate statistical significance (*p* < 0.05)

After stratifying by gender (Table 4), the CA genotype of rs67756423 was associated with a lower risk of SCZ in males (OR = 0.64, 95% CI = 0.44–0.92, *p* = 0.017). As for rs4976976, the AG and AA genotype were related to a lower incidence of SCZ under codominant (AG vs. AA, OR = 0.61, 95% CI = 0.40–0.93, *p* = 0.022) and recessive model (AA vs. AG-GG, OR = 1.53, 95% CI = 1.02–2.29, *p* = 0.041). In females, the TT genotype of rs12666575 obviously reduced the susceptibility to SCZ in recessive model (TT vs. TC-CC, OR = 0.61, 95% CI = 0.52–1.15, *p* = 0.042). Rs1107592 decreased the risk of SCZ in codominant (AG vs. GG, OR = 0.62, 95% CI = 0.40–0.95, *p* = 0.029) and dominant models (AA-AG vs. GG, OR = 0.65, 95% CI = 0.43–0.98, *p* = 0.038). However, no significant association was found after Bonferroni correction.

### MDR analysis

**Fig. 1:**
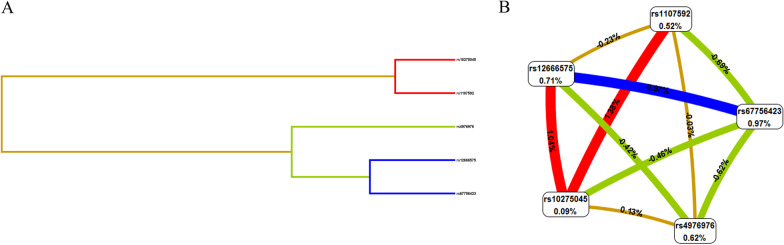
The Dendrogram and Fruchterman-Reingold of SNP-SNP interaction. **a** Dendrogram of SNP-SNP interaction. The shorter the line connecting the 2 SNPs, the stronger the interaction. **b** Fruchterman-Reingold of SNP-SNP interaction. Each SNP is reported in per cent the value of Information Gain (IG), while numbers in the connections indicate the entropy-based IG for the SNP pairs. Red bar indicates high-level synergies on the phenotype, while the brown indicates a medium-level interaction, green and blue connections with negative IG values indicate redundancy or lack of synergistic interactions between the markers

Finally, we examined the interaction of SNP-SNP using MDR. The Dendrogram and Fruchterman-Reingold of SNP-SNP interactions were exhibited in Fig. 1a and b. As is shown in Table 5, rs67756423 was the single model to forecast SCZ risk (testing accuracy = 0.509, CVC = 6/10, *p* = 0.004). The two-locus model included rs10275045 and rs1107592 (testing accuracy = 0.508, CVC = 4/10, *p* < 0.0001). The three-locus model was the combination of rs10275045, rs1107592, and rs4976976 (testing accuracy = 0.550, CVC = 10/10, *p* < 0.0001). The four-locus model comprised of rs10275045, rs1107592, rs4976976, and rs67756423 (testing accuracy = 0.548, CVC = 10/10, *p* < 0.0001). Rs10275045, rs12666575, rs1107592, rs4976976, rs67756423 were constituted five-locus model (testing accuracy = 0.522, CVC = 10/10, *p* < 0.0001). Therefore, the best model was the three-locus model, a combination of rs10275045, rs1107592, and rs4976976, with the highest testing accuracy and perfect CVC.

**Table 5:** SNP–SNP interaction models of the *MAD1L1* and *TSNARE1* genes analyzed by the MDR method

Model	Training Bal. Acc	Testing Bal. Acc	CVC	OR (95% CI)	*p*
*TSNARE1* rs67756423	0.547	0.509	6/10	1.45 (1.12–1.86)	**0.004**
*MAD1L1* rs10275045, *MAD1L1* rs1107592	0.566	0.508	4/10	2.57 (1.78–3.71)	**< 0.0001**
*MAD1L1* rs10275045, *MAD1L1* rs1107592, *TSNARE1* rs4976976	0.588	0.550	10/10	2.01 (1.56–2.59)	**< 0.0001**
*MAD1L1* rs10275045, *MAD1L1* rs1107592, *TSNARE1* rs4976976, *TSNARE1*rs67756423	0.607	0.548	10/10	2.41 (1.86–3.13)	**< 0.0001**
*MAD1L1* rs10275045, *MAD1L1* rs12666575, *MAD1L1* rs1107592, *TSNARE1* rs4976976, *TSNARE1* rs67756423	0.622	0.522	10/10	2.70 (2.09–3.51)	**< 0.0001**

MDR, multifactor dimensionality reduction; Bal. Acc., balanced accuracy; CVC, cross–validation consistency; OR, odds ratio; CI, confidence interval

*p* values were calculated using χ^2^ tests

*p* < 0.05 indicates statistical significance

### FPRP analysis

FPRP and statistical power were calculated for all positive results. As shown in Table 6, at the prior probability of 0.25 and FPRP threshold of 0.2, all significant results of *MAD1L1* and *TRNARE* polymorphisms remained noteworthy.

**Table 6:** False positive report probability of the association *MAD1L1* and *TRNARE* polymorphisms and SCZ susceptibility

Model and variables	Genotype	OR (95% CI)	*p*^a^	Statistical power	Prior probability				
					0.25	0.1	0.01	0.001	0.0001
***Overall analysis***									
rs1107592 A > G	AG vs GG	0.72 (0.54–0.97)	0.031	0.992	0.085 ^b^	0.218	0.754	0.969	0.997
rs4976976 A > G	AG vs GG	0.73 (0.54–0.99)	0.043	0.993	0.115 ^b^	0.280	0.811	0.977	0.998
rs67756423 C > A	CA vs AA	0.72 (0.55–0.94)	0.017	0.996	0.045 ^b^	0.124^b^	0.610	0.940	0.994
***Stratification analysis***									
> *36 years*									
rs12666575 T > C	TC vs CC	0.64 (0.41–1.00)	0.048	0.861	0.148 ^b^	0.343	0.852	0.983	0.998
rs1107592 A > G	AG vs GG	0.62 (0.40–0.96)	0.034	0.833	0.104 ^b^	0.258	0.792	0.975	0.997
rs4976976 A > G	AG vs GG	0.63 (0.40–0.99)	0.045	0.842	0.138 ^b^	0.325	0.841	0.982	0.998
*Male*									
rs4976976 A > G	AG vs GG	0.61 (0.40–0.93)	0.022	0.822	0.073 ^b^	0.191^b^	0.722	0.963	0.996
rs67756423 C > A	CA vs AA	0.64 (0.44–0.92)	0.017	0.909	0.050 ^b^	0.136^b^	0.635	0.946	0.994
*Female*									
rs12666575 T > C	TT vs TC + CC	0.61 (0.52–1.15)	0.042	0.731	0.342	0.609	0.945	0.994	0.999
rs1107592 A > G	AG vs GG	0.62 (0.40–0.95)	0.029	0.838	0.091 ^b^	0.232	0.769	0.971	0.997
	AA + AG vs GG	0.65 (0.43–0.98)	0.038	0.895	0.118 ^b^	0.286	0.815	0.978	0.998

SCZ, schizophrenia; OR: odds ratio; CI, confidence interval

*p*^a^ < 0.05 indicates statistical significance

^b^The level of false positive report probability threshold was set at 0.2 and noteworthy findings are presented

## Discussion

In this case–control study, our results found that rs1107592 in *MAD1*L1, rs4976976, and rs67756423 in *TSNARE* were related to a decreased risk of SCZ in the overall analysis. In addition, *MAD1L1*-rs12666575, -rs1107592, and *TSNARE-*rs4976976 significantly decreased the occurrence of SCZ individuals aged > 36 years. Subsequently, the stratification results based on age were shown that *TSNARE*-rs4976976, -rs67756423 in males and *MAD1L1*-rs1107592,-rs12666575 in females are associated with a lower risk of SCZ. These results suggested that *MAD1L1* and *TSNARE* genetic polymorphisms were associated with SCZ susceptibility and played a protective role in the development of SCZ.

*MAD1L1* is located at human chromosome 7q22.3 and involves cell cycle control and tumor suppression. Recently, some research has reported that *MAD1L1* rs12666575 was related to SCZ risk in different genetic backgrounds. For example, Sleiman et al. demonstrated that rs12666575 was associated with SCZ susceptibility in a mixed-ancestry cohorts from Caucasians, African Americans, and Asians in 2013 [15]. A genome-wide association study also discovered that rs12666575 reduced the incidence of SCZ in the Swedish sample [18]. This was consistent with our results, which found the TC and TT genotype of rs12666575 could decrease the risk of SCZ in different subgroups (age > 36 years old and women, respectively). Rs10275045 is located in the intron region of *MAD1L1*. A study showed that rs10275045 was associated with SCZ risk in European ancestry [19]. However, the relationship between rs10275045 and SCZ susceptibility was not observed in the Chinese Han population. One possible reason for the contradiction is the genetic heterogeneity of SCZ in individuals of different ethnic groups. Besides, our results revealed that the AG phenotype of rs1107592 was associated with a lower incidence of SCZ in the overall. Stratification analysis also showed that rs1107592 decreased the susceptibility to SCZ subjects with age > 36 years. And the AG and AA-AG genotype of rs1107592 played a protective role in SCZ risk of females. To the best of our knowledge, a meta-analysis study has indicated that rs1107592 was related to the susceptibility to SCZ, but OR values were not reported [16]. These results demonstrated that *MAD1L1* polymorphisms involved in the occurrence of SCZ and exerted a protective role in SCZ.

*TSNARE* is located at human chromosome 8q24.3 and may have a function in intracellular protein transport and synaptic vesicle exocytosis. Recently, the role of *TSNARE* in SCZ has attracted the attention of researchers. For example, previous analyses showed that *TSNARE* rs10098073 and rs4129585 were related to SCZ susceptibility in Caucasians [15]. Similarly, Gu et al. indicated a significant correlation between rs10098073, rs4129585 in *TSNARE* and SCZ risk in Southeast Chinese Han and Zhuang people [16]. However, there were few studies about *TSNARE* other polymorphisms and SCZ susceptibility. In the present study, our results discovered that the AG phenotype of rs4976976 and the CA genotype of rs67756423 were related to a lower risk of SCZ in the overall. Then, we further stratified analysis showed that the AG and AA genotype of rs4976976, the CA genotype of rs67756423 decreased the susceptibility to SCZ in males. Additionally, the AG phenotype of rs4976976 reduced the risk of SCZ with age > 36 years in the heterozygote model. These results indicated that *TSNARE* polymorphisms are associated with susceptibility to SCZ.

In this study, rs10275045, rs12666575, rs1107592, rs4976976 and rs67756423, located in the intron region of *MAD1L1* and *TSNARE1*, might be associated with the regulation of motifs changed, NHGRI/EBI GWAS hits, GRASP QTL Hits, selected eQTL hits, enhancer histone marks and DNAse, suggesting their potential function in SCZ. In addition, some studies provided evidence to support that intronic SNPs alter the susceptibility to diseases by regulating gene expression [20, 21]. Therefore, we speculated that *MAD1L1* and *TSNARE* polymorphisms may affect the *MAD1L1* and *TSNARE* expression to alter the risk of SCZ. However, further study is necessary to confirm this hypothesis.

This work is limited by several factors. First, the sample size of this study was relatively small. A larger sample size was needed to verify our results in future experiments. Second, only three SNPs in *MAD1L1* and two SNPs in *TSNARE* were identified in the present study, and more polymorphisms of these two genes need to be explored. Third, the clinical symptoms of patients, such as severity of disease, were missing. In the future experiments, we should collect complete clinical symptoms of patients to support our findings. Four, although *MAD1L1*/*TSNARE* polymorphisms might be associated with SCZ risk, the results were not significant after Bonferroni correction (*p* < 0.05/5). Thus, a replication experiment in a cohort with large sample size are required to confirm our findings.

## Conclusions

In conclusion, our findings suggest that genetic polymorphisms in *MAD1L1* and *TSNARE* genes may contribute to risk of SCZ in the Chinese population. These results provide evidence that *MAD1L1* and *TSNARE* may serve as potential biomarkers of SCZ. However, a replication experiment in a cohort with large sample size are required to confirm our findings.

## Supplementary Information

**Additional file 1. Supplemental table 1** Primers used in this study.

## Data Availability

The datasets generated and/or analyzed during the current study are available in the figshare repository (https://figshare.com/articles/dataset/Genotype_xlsx/14803701).

## Abbreviations

SCZ: Schizophrenia
MAD1L1: Mitotic arrest deficient-like 1
TSNARE1: T-SNARE domain-containing 1 gene
OR: Odds ratio
CI: Confidence intervals
MAF: Minor allele frequency
HWE: Hardy–Weinberg equilibrium
MDR: Multifactor dimensionality reduction
