# Supplementary material for: MAD1L1 and TSNARE gene polymorphisms are associated with schizophrenia susceptibility in the Han Chinese population
Source: BMC Med Genomics. 2021 Sep 4;14:218. doi: 10.1186/s12920-021-01070-2 (PMC8418747; doi:10.1186/s12920-021-01070-2)
Supplement: Supplementary file 1 — Additional file 1. Supplemental table 1 Primers used in this study. [file 12920_2021_1070_MOESM1_ESM.docx]

**Supplemental table 1 Primers used in this study**

| SNP | 1st-PCRP | 2nd-PCRP | UEP_SEQ |
| --- | --- | --- | --- |
| rs10275045 | ACGTTGGATGAGAATTACACGAGATGAGCC | ACGTTGGATGATGCGTGACCATTTAAACCC | TTTTGCATCTATTTCAGCCAT |
| rs12666575 | ACGTTGGATGGCCCCCATTATGCATCTTTT | ACGTTGGATGAAAGCAGGTGGAGGGTAGC | AACAAACCCATCACACA |
| rs1107592 | ACGTTGGATGACCACCGTGTTTGTGGCAG | ACGTTGGATGAGGCTCTCACACCTGCTTTG | AGTGACATGTTTGCCGCA |
| rs4976976 | ACGTTGGATGCTCCCTCCTTTGGATACTTG | ACGTTGGATGGCAAGGGATTAAGGGAGAAC | GAAAAACGGGAGCTTAAAT |
| rs67756423 | ACGTTGGATGTTCTCTGTCTGCAGGGCAC | ACGTTGGATGAAACCAAACAAACCGCCCAG | AGGGCACGCACGTTGAG |

SNP, single-nucleotide polymorphism; PCRP, primer for polymerase chain reaction; UEP_SEQ, primer for single nucleotide extension.
